# Detailed Knowledge About HIV Epidemiology and Transmission Dynamics and Their Associations With Preventive and Risk Behaviors Among Gay, Bisexual, and Other Men Who Have Sex With Men in the United States

**DOI:** 10.2196/publichealth.7255

**Published:** 2017-03-06

**Authors:** Akshay Sharma, Stephen P Sullivan, Rob B Stephenson

**Affiliations:** ^1^ Center for Sexuality and Health Disparities Department of Health Behavior and Biological Sciences University of Michigan School of Nursing Ann Arbor, MI United States

**Keywords:** HIV infections, sexual minorities, social networking, risk reduction behavior, sexual behavior

## Abstract

**Background:**

Gay, bisexual, and other men who have sex with men (GBMSM) in the United States remain disproportionately affected by human immunodeficiency virus (HIV). Yet their testing frequency is suboptimal and condomless anal sex (CAS) is increasing. Behavioral theories posit that information about HIV is a pivotal construct in individual risk reduction. However, measurements of knowledge have traditionally focused on ever hearing about HIV and being aware of the most common routes of spread.

**Objective:**

Using a national Web-based sample of sexually active GBMSM, we sought to (1) quantify levels of detailed knowledge about HIV epidemiology and transmission dynamics, (2) describe variations in detailed knowledge levels across demographic strata, and (3) evaluate potential associations of increasing levels of detailed knowledge with HIV testing in the past year and engaging in CAS with a male partner in the past 3 months.

**Methods:**

GBMSM were recruited through a social networking website (Facebook) from August to September 2015 and asked 17 knowledge-based questions pertaining to the following 2 domains using a Web-based survey: HIV epidemiology (9 questions including statistics on incidence, prevalence, and distribution) and HIV transmission dynamics (8 questions including modes of spread and per-act transmission probabilities). Ordinal domain-specific indices of detailed knowledge were created for each respondent by summing their number of correct responses. Separate cumulative logit models were used to identify factors independently associated with each index, and multivariable logistic regression models were used to characterize associations with HIV testing history and recently engaging in CAS.

**Results:**

Of the 1064 participants in our study, only half (49.62%, 528/1064) had been tested for HIV in the past year, and almost half (47.84%, 509/1064) had engaged in CAS with a male partner in the past 3 months. Majority scored 3 of 9 epidemiology questions correct (26.88%, 286/1064) and 5 of 8 transmission dynamics questions correct (25.00%, 266/1064). Participants younger than 35 years, of non-Hispanic non-white or Hispanic race and ethnicity, with lower educational levels, and who reported a sexual orientation other than homosexual or gay were significantly less knowledgeable about HIV transmission dynamics. Increasing levels of knowledge about this domain were independently associated with testing in the past year (adjusted odds ratio for each additional correct response: 1.10, 95% CI 1.01-1.20) but not with recent CAS. Increasing knowledge about HIV epidemiology was not associated with either outcome.

**Conclusions:**

Increasing detailed knowledge about HIV epidemiology might not be as important as educating sexually active GBMSM regarding transmission dynamics. Researchers and practitioners designing prevention messages targeting GBMSM should bear in mind that not all knowledge is equal and that some aspects might have a greater positive impact than others. Future research to identify influential content and contemporary modes of delivery is needed.

## Introduction

Gay, bisexual, and other men who have sex with men (GBMSM) continue to bear the greatest burden of human immunodeficiency virus (HIV) in the United States. From 2005 to 2014, HIV diagnoses in this risk group increased by 6%, driven predominantly by increases among non-Hispanic black or African American and Hispanic GBMSM [1]. Two-thirds (67%) of the total estimated 44,073 new diagnoses in 2014 were attributable to male-to-male sexual contact [2]. According to a recent analysis, 15% of the approximately 4.5 million GBMSM in the United States are living with HIV, and the prevalence in this community is estimated to be as high as 57.5 times that of other US men [3]. Given these trends, allocating resources toward high-impact HIV prevention services for GBMSM and improving outcomes at every step of the care continuum have been recognized as national priorities by the White House [4].

Despite significant advances in our understanding of biomedical approaches to reduce HIV transmission, such as preexposure prophylaxis (PrEP) [5], actual utilization among high-risk individuals has been suboptimal. Data from the 2014 National HIV Behavioral Surveillance (NHBS) system indicate that only 3% of 6847 HIV-negative GBMSM reported taking PrEP in the preceding year [6]. Mathematical modeling suggests that 40% coverage among GBMSM indicated for PrEP could avert up to 33% of incident HIV infections over the next 10 years [7]. Treatment as prevention (TasP) is another strategy that has been shown to reduce sexual transmission from people living with HIV by more than 96% [8]. Although there have been recent improvements in antiretroviral therapy prescription and viral suppression among GBMSM, racial and ethnic disparities persist [9].

HIV testing itself may be considered an important prevention activity, as it is the critical first step in accessing prophylactic services among at-risk individuals [10] and for initiating antiretroviral therapy among those who are living with HIV. Unfortunately, many GBMSM in the United States do not test annually, as recommended by the Centers for Disease Control and Prevention (CDC) [11]. Almost a third (29%) of 8243 GBMSM participating in the 2014 NHBS reported not having been tested in the past year, and a quarter (25%) of 1888 GBMSM living with HIV were unaware of their serostatus [6]. Perhaps even more troubling is the increasing prevalence of high-risk sexual behavior in this community. The overall proportions of GBMSM who reported engaging in condomless anal sex (CAS) with a male partner within 12 months preceding their NHBS interview were 48% in 2005, 54% in 2008, 57% in 2011, and 64% in 2014 [6,12].

Interventions to prevent HIV transmission have been guided by several theoretical approaches, including the information-motivation-behavioral skills model [13], the AIDS risk reduction model [14], the transtheoretical model of behavior change [15], the health belief model [16], and social cognitive theory [17]. According to the information-motivation-behavioral skills model for individual-level changes in HIV risk behavior, risk reduction can be conceptualized as a function of people’s information about HIV, their motivation to reduce risk, and their behavioral skills to successfully undertake specific prevention activities. A pivotal construct of this model is information, which refers generally to an individual’s knowledge about HIV that might include how the disease develops, its expected course, and effective strategies for its prevention and management [18].

Although knowledge around HIV has been the cornerstone of public health research throughout the epidemic, measurements of knowledge have traditionally focused on whether one has ever heard about HIV [19] and being aware of the most common routes of spread [20,21]. Surveys seeking to measure multiple dimensions of HIV awareness have also been developed for college students. For example, the Attitudes Toward AIDS Scale uses a true-false format to tap basic knowledge about prevalence, medical facts, misconceptions, transmission, and prevention methods [22], and the International AIDS Questionnaire uses a 5-point Likert item format to capture increasing levels of agreement with myths and facts about transmission and statements evaluating attitudes toward people living with HIV [23]. However, we are not aware of previous research that has assessed the extent of detailed knowledge about HIV epidemiology and transmission dynamics specifically among GBMSM in the United States and how these domains might relate to preventive and high-risk sexual behaviors.

Using a large national sample of sexually active GBMSM, we sought to quantify levels of detailed HIV knowledge (such as publicly available statistics on disease burden and distribution and per-act transmission probabilities) and to describe variations across strata of demographic characteristics. Furthermore, we sought to evaluate potential associations of increasing levels of detailed knowledge about HIV epidemiology and transmission dynamics with HIV testing history and with recently engaging in CAS with a male partner. Understanding these issues can help guide future HIV education and prevention efforts in the United States, particularly among disproportionately affected subgroups of GBMSM.

## Methods

Participants were recruited through selective placement of banner advertisements featured on a social networking website (Facebook from August to September 2015. Recruitment was targeted toward user profiles that were male, 18 years of age or older, resided within the United States, and reflected a variety of gay or bisexual-related interests. Within the advertising management software, “interests” represent topical categories of information that users have accessed through posts on their timeline, keywords from pages they have liked, as well as advertisements they have clicked on previously. In our case, potential participants’ interests could have been broad in scope (eg, lesbian, gay, bisexual, and transgender community, same-sex marriage) or specific (eg, BuzzFeed LGBT, Out Magazine). Rather than focusing solely on men who indicated they were interested in other men in their Facebook profiles, interest-based targeting allowed for the possibility of recruiting GBMSM who were not out to their families or friends. The advertisements included images of men kissing or holding hands, the survey title (“The Prioritizing U survey: Tell us what matters”), as well as the following call-to-action text: “Help the University of Michigan understand what’s important to you and your health.” Individuals who clicked through the banner advertisements were directed to a Web-based informed consent module, and those who consented were screened to determine eligibility before being administered a Web-based survey. The eligibility criteria for the study included reporting a male gender identity, being 18 years of age or older, currently residing within the United States, and having sex with a male partner in the past 6 months. No monetary incentives were provided to the participants for completing our survey. Ethical approval for this study was obtained from the University of Michigan’s Institutional Review Board.

Demographic information collected from all participants included their age, race and ethnicity, educational level, employment status, sexual orientation, relationship status, and state of residence. Participants were also asked to report the number of people that they personally knew who were living with HIV and those who had died of HIV-related complications. Several questions were used to elicit information on participants’ HIV testing history and recent potentially high-risk sexual behavior. Responses for “Have you ever been tested for HIV, the retrovirus that causes AIDS?” and “When were you last tested for HIV?” were combined to construct a dichotomous variable: tested for HIV in the past year versus not. Data on the number of male anal sex partners in the past 3 months, sexual positioning (insertive or receptive role), and condom use during each encounter were used to construct another dichotomous variable: engaged in CAS in the past 3 months versus not. Finally, participants were asked a series of 17 knowledge-based questions pertaining to the following 2 domains: HIV epidemiology (9 multiple-choice questions including statistics on the incidence, prevalence, and distribution in different populations) and HIV transmission dynamics (8 multiple-choice questions including common modes of spread and per-act transmission probabilities). These domains were selected because their factual details are readily available to the public on CDC’s HIV website. Our knowledge-based questions along with their response options are presented in Multimedia Appendices 1 and (HIV epidemiology and HIV transmission dynamics, respectively). For analytical purposes, ordinal domain-specific indices of detailed knowledge were created for each respondent by summing their number of correct responses, in a manner similar to previous studies [24,25]. Given their ordinal nature, the Spearman rank correlation coefficient was calculated to assess any potential relationship between the 2 indices.

Statistical analyses were conducted using SAS version 9.4 (SAS Institute Inc). Because of our focus on whether detailed HIV knowledge influences preventive and risky sexual behaviors among at-risk GBMSM, we restricted our analyses to participants who reported being HIV-negative or of unknown status, had anal sex with a male partner in the past 3 months, and answered all knowledge-based questions. Selected demographic and behavioral characteristics were summarized using descriptive statistics. The proportions of participants, who reported testing for HIV in the past year and engaging in CAS in the past 3 months, were estimated within strata of response validity for each knowledge-based question. Separate cumulative logit models were used to identify factors independently associated with each of the ordinal domain-specific indices of detailed knowledge. Finally, 2 multivariable logistic regression models were used to characterize associations of domain-specific knowledge levels with HIV testing and with recently engaging in CAS, after controlling for several potential confounders. Each model included the following covariates: detailed HIV epidemiology knowledge (ordinal), detailed HIV transmission dynamics knowledge (ordinal), age group (18-34, 35-54, ≥55 years), race and ethnicity (non-Hispanic white, non-Hispanic non-white, Hispanic), educational level (associate's or technical degree or lower, bachelor’s degree, master’s or doctoral degree), employment status (work full-time, work part-time, other), sexual orientation (homosexual or gay, other), relationship status (single, partnered: monogamous, partnered: open relationship), region (West, Midwest, Northeast, South), know people living with HIV (no, yes: 1-2 people, yes: ≥3 people), and know people who died of HIV-related complications (no, yes: 1-2 people, yes: ≥3 people). Assessments for potential multicollinearity issues between explanatory variables were conducted by examining condition indices and variance decomposition proportions [26]. Results from all models are presented as adjusted odds ratios with their 95% CIs.

## Results

**Figure 1:**
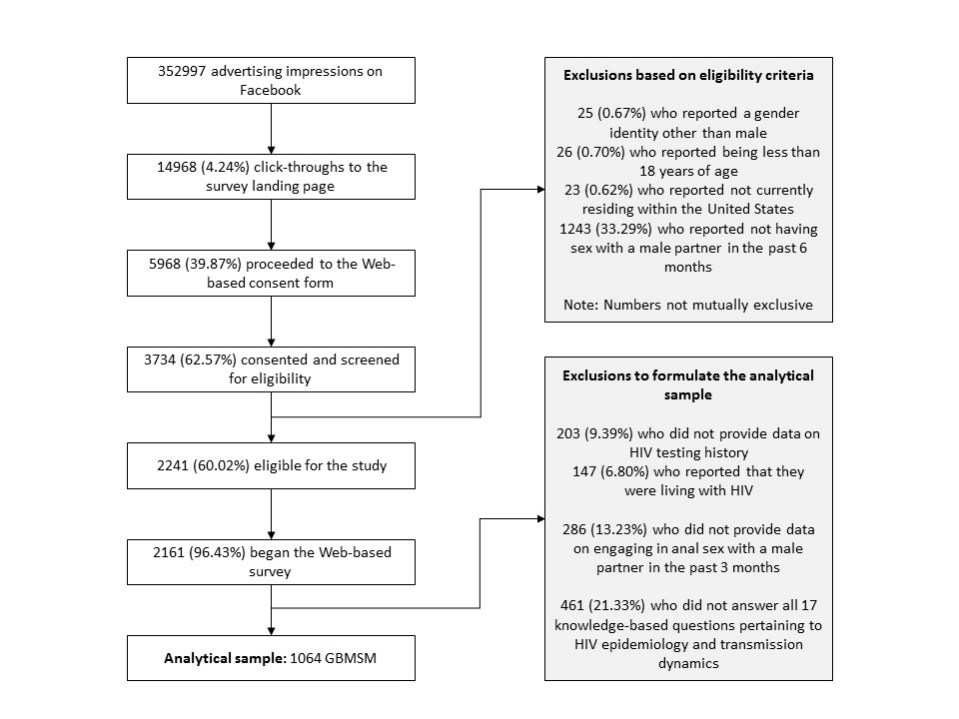
Formulation of the analytical sample of 1064 HIV-negative or unknown status gay, bisexual, and other men who have sex with men (GBMSM), United States, August-September 2015.

Overall, 352,997 advertising impressions resulted in 14,968 click-throughs to the survey landing page over a 4-week period. Of these, 5968 proceeded to the Web-based consent form, and 3734, who provided consent, were screened for eligibility. Our final analytical sample was restricted to 1064 of 2161 eligible study participants who provided data on their HIV testing history, reported they were not living with HIV, volunteered information on engaging in anal sex with a male partner in the past 3 months, and answered all 17 knowledge-based questions pertaining to HIV epidemiology and transmission dynamics (see Figure 1). Excluded individuals were more likely to have lower levels of education and to report an orientation other than homosexual or gay but were similar with respect to other demographic characteristics.

Table 1 summarizes the descriptive characteristics of 1064 participants included in our analyses. Majority of the participants were middle-aged (mean 45 years, median 49 years), were non-Hispanic white, had an associate’s or technical degree or lower educational level, and worked full-time. Almost two-thirds (60.34%, 642/1064) reported having a primary male partner (described as “Someone you feel committed to above all others. You might call this person your boyfriend, partner, significant other, spouse, or husband.”). More than a third (38.82%, 413/1064) reported personally knowing ≥3 people living with HIV, and a third (32.99%, 351/1064) reported knowing ≥3 people who died of HIV-related complications.

Regarding participants’ HIV testing history, half (49.62%, 528/1064) reported having been tested in the past year, 317 (60.04%) of whom had been tested in the past 6 months. Of the 1064 participants, 136 (12.78%) had never been tested for HIV. Additionally, almost half (47.84%, 509/1064) of our sample reported engaging in CAS in the past 3 months. Of these 509 participants, 321 (63.06%) had CAS with 1 man, 49 (9.63%) had CAS with 2 men, and 139 (27.31%) had CAS with ≥3 men. Regarding sexual positioning, 221 (43.42%) engaged in both insertive and receptive CAS, 137 (26.92%) engaged in only insertive CAS, and 151 (29.67%) engaged in only receptive CAS.

**Table 1:** Descriptive characteristics of 1064 HIV-negative or unknown status gay, bisexual, and other men who have sex with men, United States, August-September 2015.

Characteristic		Frequency and percentage distribution, n (%)	Tested for HIV^a^ in the past year (n=528), n (%)^b^	Engaged in CAS^c^ in the past 3 months (n=509), n (%)^d^
**Age group, years^e^**				
	18-34	315 (29.6)	184 (58.4)	188 (59.7)
	35-54	435 (40.9)	212 (48.7)	212 (48.7)
	≥55	314 (29.5)	132 (42.0)	109 (34.7)
**Race and ethnicity**				
	Non-Hispanic white	852 (80.1)	387 (45.4)	411 (48.2)
	Non-Hispanic non-white^f^	94 (8.8)	57 (60.6)	42 (44.7)
	Hispanic	118 (11.1)	84 (71.2)	56 (47.5)
**Educational level**				
	Associate's or technical degree or lower^g^	457 (43.0)	223 (48.8)	224 (49.0)
	Bachelor’s degree	336 (31.6)	161 (47.9)	164 (48.8)
	Master’s or doctoral degree	271 (25.5)	144 (53.1)	121 (44.6)
**Employment status**				
	Work full-time	778 (73.1)	390 (50.1)	397 (51.0)
	Work part-time	121 (11.4)	63 (52.1)	59 (48.8)
	Other^h^	165 (15.5)	75 (45.5)	53 (32.1)
**Sexual orientation**				
	Homosexual or gay	897 (84.3)	453 (50.5)	451 (50.3)
	Other^i^	167 (15.7)	75 (44.9)	58 (34.7)
**Relationship status**				
	Single	422 (39.7)	223 (52.8)	139 (32.9)
	Partnered: monogamous^j^	462 (43.4)	185 (40.0)	274 (59.3)
	Partnered: open relationship^k^	180 (16.9)	120 (66.7)	96 (53.3)
**Region**				
	West	232 (21.8)	134 (57.8)	108 (46.6)
	Midwest	269 (25.3)	118 (43.9)	133 (49.4)
	Northeast	195 (18.3)	83 (42.6)	93 (47.7)
	South	368 (34.6)	193 (52.4)	175 (47.6)
**Know people living with HIV**				
	No	356 (33.5)	143 (40.2)	143 (40.2)
	Yes: 1-2 people	295 (27.7)	143 (48.5)	158 (53.6)
	Yes: ≥3 people	413 (38.8)	242 (58.6)	208 (50.4)
**Know people who died of HIV-related complications**				
	No	458 (43.0)	244 (53.3)	242 (52.8)
	Yes: 1-2 people	255 (24.0)	119 (46.7)	130 (51.0)
	Yes: ≥3 people	351 (33.0)	165 (47.0)	137 (39.0)

^a^HIV: human immunodeficiency virus.

^b^Percentages indicate the proportion who had been tested for HIV in the past year within each stratum of demographic and behavioral characteristics.

^c^CAS: condomless anal sex.

^d^Percentages indicate the proportion who engaged in CAS in the past 3 months within each stratum of demographic and behavioral characteristics.

^e^Age: mean 45 years, median 49 years, range 18-87 years.

^f^Includes 30 non-Hispanic black or African American, 18 Asian, 12 Native American or Alaskan Native, 2 Native Hawaiian or Pacific Islander, and 32 other.

^g^Includes 110 with an associate's or technical degree, 261 with some college education, 77 with a high school diploma or General Educational Development (GED), and 9 with some high school education.

^h^Includes 78 retired, 30 unemployed, 26 who are collecting disability, 18 students, and 13 other.

^i^Includes 139 bisexual, 10 heterosexual or straight, 13 questioning or unsure, 2 queer, and 3 other.

^j^Described as “You and your partner are exclusively having sex with one another.”

^k^Includes 150 in an open relationship with restrictions (described as “You and your partner are allowed to have sex with other people but under certain rules.”) and 30 in an open relationship without restrictions (described as “You and your partner are allowed to have sex with other people without rules.”).

Individual questions assessing participants’ detailed knowledge about HIV epidemiology and transmission dynamics (with answers) are presented in Tables 2 and 3, respectively. The proportion of correct responses selected for questions pertaining to epidemiology ranged from 20.86% (for question 8) to 57.33% (for question 9). Majority (26.88%, 286/1064) of the participants scored 3 of 9 epidemiology questions correct (median 3, range 0-8). The proportion of correct responses selected for questions pertaining to transmission dynamics ranged from 21.05% (for question 4) to 68.98% (for question 5). Majority (25.00%, 266/1064) of the participants scored 5 of 8 transmission dynamics questions correct (median 4, range 0-7). Similar proportions reported testing for HIV in the past year and engaging in CAS in the past 3 months within each stratum of response validity for almost all questions. The 2 domain-specific indices of detailed knowledge were not correlated (Spearman rank correlation coefficient=.03, *P*=.28).

**Table 2:** Detailed knowledge about HIV epidemiology among 1064 HIV-negative or unknown status gay, bisexual, and other men who have sex with men, United States, August-September 2015.

Questions assessing detailed knowledge about HIV^a^ epidemiology (correct answer)		Validity of selected response, n (%)	Tested for HIV in the past year (n=528), n (%)^b^	Engaged in CAS^c^ in the past 3 months (n=509), n (%)^d^
**1. Approximately how many people in the United States are newly infected with HIV each year? (50,000)**				
	Correct	250 (23.5)	124 (49.6)	115 (46.0)
	Incorrect	814 (76.5)	404 (49.6)	394 (48.4)
**2. Approximately how many people in the United States are currently living with HIV? (1.2 million)**				
	Correct	531 (49.9)	264 (49.7)	256 (48.2)
	Incorrect	533 (50.1)	264 (49.5)	253 (47.5)
**3. What percentage of all those living with HIV in the United States do not know that they are infected? (14%)**				
	Correct	287 (27.0)	140 (48.8)	134 (46.7)
	Incorrect	777 (73.0)	388 (49.9)	375 (48.3)
**4. Even though men who have sex with men comprise only 2% of the United States population, what proportion of new HIV infections did they account for annually from 2008-2010? (3/4)**				
	Correct	302 (28.4)	154 (51.0)	150 (49.7)
	Incorrect	762 (71.6)	374 (49.1)	359 (47.1)
**5. Even though African Americans comprise only 12% of the United States population, what percentage of new HIV infections did they account for in 2010? (44%)**				
	Correct	427 (40.1)	210 (49.2)	214 (50.1)
	Incorrect	637 (59.9)	318 (49.9)	295 (46.3)
**6. Even though young people aged 13-24 years comprise only 16% of the United States population, what percentage of new HIV infections did they account for in 2010? (26%)**				
	Correct	506 (47.6)	257 (50.8)	243 (48.0)
	Incorrect	558 (52.4)	271 (48.6)	266 (47.7)
**7. Who had the highest percentage of newly identified HIV-positive test results in 2010? (Transgender individuals or those who do not identify with their assigned sex at birth)**				
	Correct	230 (21.6)	112 (48.7)	123 (53.5)
	Incorrect	834 (78.4)	416 (49.9)	386 (46.3)
**8. Specifically among young people aged 13-24 years, what percentage of all new HIV infections did young men who have sex with men account for in 2010? (72%)**				
	Correct	222 (20.9)	116 (52.3)	116 (52.3)
	Incorrect	842 (79.1)	412 (48.9)	393 (46.7)
**9. Among gay and bisexual men in 2013, _____ accounted for the largest estimated percentage of HIV diagnoses followed by _____ and _____. (African Americans, Whites, Hispanics or Latinos)**				
	Correct	610 (57.3)	324 (53.1)	305 (50.0)
	Incorrect	454 (42.7)	204 (44.9)	204 (44.9)

^a^HIV: human immunodeficiency virus.

^b^Percentages indicate the proportion who had been tested for HIV in the past year within each stratum of response validity.

^c^CAS: condomless anal sex.

^d^Percentages indicate the proportion who engaged in CAS in the past 3 months within each stratum of response validity.

**Table 3:** Detailed knowledge about HIV transmission dynamics among 1064 HIV-negative or unknown status gay, bisexual, and other men who have sex with men, United States, August-September 2015.

Questions assessing detailed knowledge about HIV^a^ transmission dynamics (correct answer)	Validity of selected response, n (%)	Tested for HIV in the past year (n=528), n (%)^b^	Engaged in CAS^c^ in the past 3 months (n=509), n (%)^d^
**1. The estimated probability of HIV infection per-act of receptive anal intercourse (receiving the penis into the anus, also known as bottoming) without any means of prevention is _____ per 10,000 exposures. (138)**			
Correct	486 (45.7)	254 (52.3)	233 (47.9)
Incorrect	578 (54.3)	274 (47.4)	276 (47.8)
**2. The estimated probability of HIV infection per-act of insertive anal intercourse (inserting the penis into the anus, also known as topping) without any means of prevention is _____ per 10,000 exposures. (11)**			
Correct	493 (46.3)	252 (51.1)	238 (48.3)
Incorrect	571 (53.7)	276 (48.3)	271 (47.5)
**3. The estimated probability of HIV infection per-act of receptive vaginal intercourse (receiving the penis into the vagina) without any means of prevention is _____ per 10,000 exposures. (8)**			
Correct	348 (32.7)	170 (48.9)	160 (46.0)
Incorrect	716 (67.3)	358 (50.0)	349 (48.7)
**4. The estimated probability of HIV infection per-act of insertive vaginal intercourse (inserting the penis into the vagina) without any means of prevention is _____ per 10,000 exposures. (4)**			
Correct	224 (21.1)	106 (47.3)	105 (46.9)
Incorrect	840 (78.9)	422 (50.2)	404 (48.1)
**5. The estimated probability of acquiring HIV from kissing without any means of prevention is _____ per 10,000 exposures. (0)**			
Correct	734 (69.0)	379 (51.6)	366 (49.9)
Incorrect	330 (31.0)	149 (45.2)	143 (43.3)
**6. The estimated probability of acquiring HIV from needle-sharing during injection drug use without any means of prevention is _____ per 10,000 exposures. (63)**			
Correct	707 (66.4)	359 (50.8)	343 (48.5)
Incorrect	357 (33.6)	169 (47.3)	166 (46.5)
**7. The estimated probability of acquiring HIV from a mosquito bite without any means of prevention is _____ per 10,000 exposures. (0)**			
Correct	653 (61.4)	338 (51.8)	310 (47.5)
Incorrect	411 (38.6)	190 (46.2)	199 (48.4)
**8. What is the probability of acquiring HIV from receptive or insertive oral sex without using any means of prevention? (Extremely low)**			
Correct	676 (63.5)	345 (51.0)	337 (49.9)
Incorrect	388 (36.5)	183 (47.2)	172 (44.3)

^a^HIV: human immunodeficiency virus.

^b^Percentages indicate the proportion who had been tested for HIV in the past year within each stratum of response validity.

^c^CAS: condomless anal sex.

^d^Percentages indicate the proportion who engaged in CAS in the past 3 months within each stratum of response validity.

Table 4 presents results from our cumulative logit models used to identify factors independently associated with each of the domain-specific indices of detailed knowledge. Regarding detailed HIV epidemiology knowledge, participants with a master’s or doctoral degree were significantly more likely to have higher knowledge levels compared with those with an associate’s or technical degree or lower educational level. However, relative to participants residing in the West of the United States, those from the Midwest and the Northeast were significantly less knowledgeable about this domain. Regarding detailed HIV transmission dynamics knowledge, participants aged ≥35 years were significantly more likely to have higher knowledge levels compared with those who were younger. Similar findings were observed for participants with a bachelor’s degree or higher educational level versus a lower educational level and for those who personally knew ≥3 people living with HIV versus none. However, non-Hispanic non-white and Hispanic participants were significantly less knowledgeable about HIV transmission dynamics compared with non-Hispanic whites. Finally, relative to participants who reported being homosexual or gay, those who reported some other sexual orientation were also significantly less knowledgeable about this domain.

**Table 4:** Factors independently associated with detailed knowledge about HIV epidemiology and transmission dynamics among 1064 HIV-negative or unknown status gay, bisexual, and other men who have sex with men, United States, August-September 2015.

Characteristic		Detailed HIV^a^ epidemiology knowledge^b^ aOR^c^ (95% CI)	Detailed HIV transmission dynamics knowledge^d^ aOR (95% CI)
**Age group, years^e^**			
	18-34	Reference	Reference
	35-54	0.79 (0.59-1.07)	1.48 (1.10-2.01)^f^
	≥55	0.70 (0.50-1.00)	1.56 (1.10-2.21)^f^
**Race and ethnicity**			
	Non-Hispanic white	Reference	Reference
	Non-Hispanic non-white^g^	1.11 (0.75-1.63)	0.63 (0.43-0.92)^f^
	Hispanic	0.90 (0.63-1.28)	0.70 (0.49-0.99)^f^
**Educational level**			
	Associate's or technical degree or lower^h^	Reference	Reference
	Bachelor’s degree	1.01 (0.78-1.30)	1.74 (1.35-2.25)^f^
	Master’s or doctoral degree	1.36 (1.04-1.79)^f^	2.41 (1.82-3.17)^f^
**Employment status**			
	Work full-time	Reference	Reference
	Work part-time	0.72 (0.50-1.04)	0.80 (0.55-1.14)
	Other^i^	0.73 (0.53-1.00)	1.06 (0.77-1.45)
**Sexual orientation**			
	Homosexual or gay	Reference	Reference
	Other^j^	0.82 (0.60-1.13)	0.57 (0.41-0.78)^f^
**Relationship status**			
	Single	Reference	Reference
	Partnered: monogamous^k^	1.00 (0.78-1.28)	0.90 (0.70-1.15)
	Partnered: open relationship^l^	1.34 (0.97-1.85)	0.99 (0.72-1.36)
**Region**			
	West	Reference	Reference
	Midwest	0.71 (0.52-0.97)^f^	0.86 (0.63-1.18)
	Northeast	0.71 (0.50-0.99)^f^	0.80 (0.57-1.13)
	South	0.91 (0.68-1.22)	0.95 (0.71-1.28)
**Know people living with HIV**			
	No	Reference	Reference
	Yes: 1-2 people	0.89 (0.67-1.17)	1.11 (0.83-1.47)
	Yes: ≥3 people	0.95 (0.71-1.27)	1.76 (1.31-2.36)^f^
**Know people who died of HIV-related complications**			
	No	Reference	Reference
	Yes: 1-2 people	0.85 (0.63-1.15)	0.98 (0.73-1.32)
	Yes: ≥3 people	0.95 (0.69-1.31)	0.80 (0.58-1.10)

^a^HIV: human immunodeficiency virus.

^b^Ordinal index created by summing the number of correct responses on questions pertaining to HIV epidemiology. Majority (26.88%, 286/1064) of participants scored 3 of 9 questions correct (median 3, range 0-8).

^c^aOR: adjusted odds ratio.

^d^Ordinal index created by summing the number of correct responses on questions pertaining to HIV transmission dynamics. Majority (25.00%, 266/1064) of participants scored 5 of 8 questions correct (median 4, range 0-7).

^e^Age: mean 45 years, median 49 years, range 18-87 years.

^f^These results indicate a statistically significant association.

^g^Includes 30 non-Hispanic black or African American, 18 Asian, 12 Native American or Alaskan Native, 2 Native Hawaiian or Pacific Islander, and 32 other.

^h^Includes 110 with an associate's or technical degree, 261 with some college education, 77 with a high school diploma or General Educational Development (GED), and 9 with some high school education.

^i^Includes 78 retired, 30 unemployed, 26 who are collecting disability, 18 students, and 13 other.

^j^Includes 139 bisexual, 10 heterosexual or straight, 13 questioning or unsure, 2 queer, and 3 other.

^k^Described as “You and your partner are exclusively having sex with one another.”

^l^Includes 150 in an open relationship with restrictions (described as “You and your partner are allowed to have sex with other people but under certain rules.”) and 30 in an open relationship without restrictions (described as “You and your partner are allowed to have sex with other people without rules.”).

Results from our multivariable logistic regression models used to characterize associations of domain-specific knowledge levels with HIV testing in the past year and with engaging in CAS in the past 3 months are summarized in Table 5. After adjusting for other characteristics, increasing levels of detailed knowledge about HIV epidemiology were not significantly associated with either of these analytical outcomes. However, increasing levels of detailed knowledge about HIV transmission dynamics were significantly associated with HIV testing in the past year. For each additional correct response to questions in this domain, the adjusted odds of reported testing increased by 10%. Finally, increasing levels of detailed knowledge about HIV transmission dynamics were not significantly associated with recently engaging in CAS.

**Table 5:** Factors independently associated with testing for HIV in the past year and engaging in condomless anal sex in the past 3 months among 1064 HIV-negative or unknown status gay, bisexual, and other men who have sex with men, United States, August-September 2015.

Characteristic		Tested for HIV^a^ in the past year aOR^b^ (95% CI)	Engaged in CAS^c^ in the past 3 months aOR (95% CI)
Detailed HIV epidemiology knowledge^d^		1.01 (0.93-1.11)	1.06 (0.97-1.17)
Detailed HIV transmission dynamics knowledge^e^		1.10 (1.01-1.20)^f^	1.05 (0.96-1.15)
**Age group, years^g^**			
	18-34	Reference	Reference
	35-54	0.53 (0.37-0.77)^f^	0.46 (0.32-0.67)^f^
	≥55	0.44 (0.29-0.67)^f^	0.33 (0.22-0.52)^f^
**Race and ethnicity**			
	Non-Hispanic white	Reference	Reference
	Non-Hispanic non-white^h^	1.81 (1.13-2.90)^f^	0.82 (0.51-1.30)
	Hispanic	2.67 (1.70-4.18)^f^	0.82 (0.54-1.26)
**Educational level**			
	Associate’s or technical degree or lower^i^	Reference	Reference
	Bachelor’s degree	0.92 (0.67-1.26)	0.76 (0.56-1.04)
	Master’s or doctoral degree	1.20 (0.85-1.68)	1.77 (0.54-1.08)
**Employment status**			
	Work full-time	Reference	Reference
	Work part-time	1.06 (0.69-1.65)	0.90 (0.58-1.40)
	Other^j^	1.04 (0.71-1.53)	0.55 (0.37-0.83)^f^
**Sexual orientation**			
	Homosexual or gay	Reference	Reference
	Other^k^	0.93 (0.63-1.36)	0.81 (0.54-1.20)
**Relationship status**			
	Single	Reference	Reference
	Partnered: monogamous^l^	0.57 (0.42-0.76)^f^	3.10 (2.30-4.19)^f^
	Partnered: open relationship^m^	1.79 (1.21-2.66)^f^	2.34 (1.59-3.45)^f^
**Region**			
	West	Reference	Reference
	Midwest	0.59 (0.40-0.87)^f^	1.11 (0.76-1.63)
	Northeast	0.61 (0.40-0.92)^f^	1.10 (0.73-1.67)
	South	0.84 (0.59-1.20)	1.02 (0.71-1.45)
**Know people living with HIV**			
	No	Reference	Reference
	Yes: 1-2 people	1.59 (1.13-2.24)^f^	2.05 (1.45-2.91)^f^
	Yes: ≥3 people	3.05 (2.12-4.40)^f^	2.27 (1.57-3.29)^f^
**Know people who died of HIV-related complications**			
	No	Reference	Reference
	Yes: 1-2 people	0.84 (0.59-1.21)	0.99 (0.69-1.42)
	Yes: ≥3 people	0.68 (0.46-1.01)	0.63 (0.42-0.93)^f^

^a^HIV: human immunodeficiency virus.

^b^aOR: adjusted odds ratio.

^c^CAS: condomless anal sex.

^d^Ordinal index created by summing the number of correct responses on questions pertaining to HIV epidemiology. Majority (26.88%, 286/1064) of participants scored 3 of 9 questions correct (median 3, range 0-8).

^e^Ordinal index created by summing the number of correct responses on questions pertaining to HIV transmission dynamics. Majority (25.00%, 266/1064) of participants scored 5 of 8 questions correct (median 4, range 0-7).

^f^These results in italics indicate a statistically significant association.

^g^Age: mean 45 years, median 49 years, range 18-87 years.

^h^Includes 30 non-Hispanic black or African American, 18 Asian, 12 Native American or Alaskan Native, 2 Native Hawaiian or Pacific Islander, and 32 other.

^i^Includes 110 with an associate's or technical degree, 261 with some college education, 77 with a high school diploma or General Educational Development (GED), and 9 with some high school education.

^j^Includes 78 retired, 30 unemployed, 26 who are collecting disability, 18 students, and 13 other.

^k^Includes 139 bisexual, 10 heterosexual or straight, 13 questioning or unsure, 2 queer, and 3 other.

^l^Described as “You and your partner are exclusively having sex with one another.”

^m^Includes 150 in an open relationship with restrictions (described as “You and your partner are allowed to have sex with other people but under certain rules.”) and 30 in an open relationship without restrictions (described as “You and your partner are allowed to have sex with other people without rules.”).

## Discussion

### Principal Findings

Our study found substantial variations in the levels of detailed HIV knowledge across strata of Web-using GBMSM in the United States. Demographic differences were more pronounced in the knowledge domain of HIV transmission dynamics compared with HIV epidemiology. Younger participants, racial and ethnic minorities, those with a lower educational level, those who did not identify as homosexual or gay, and those who personally knew fewer than 3 people living with HIV were significantly less knowledgeable about HIV transmission dynamics. In light of our result that increasing levels of detailed knowledge about this domain were positively associated with testing for HIV in the past year, directing prevention education efforts toward subgroups of GBMSM that might be currently underserved could help improve testing behavior. However, the lack of an association with engaging in CAS in the past 3 months in our sample suggests that increasing HIV transmission dynamics knowledge might not be sufficient in reducing risky sexual behavior. Our results also suggest that educating GBMSM about specific details regarding HIV epidemiology might not confer a public health benefit.

PrEP and TasP are promising biomedical strategies for preventing incident HIV infections among high-risk persons [8,10]. HIV testing is the gateway to accessing these services, and regular testing among at-risk populations needs to be prioritized. Current national recommendations state that all sexually active GBMSM should be tested for HIV at least annually [11], and those who have multiple partners or use illicit drugs concurrent with sexual activity might benefit from more frequent screening, such as every 3 to 6 months [27]. The fact that only half of our sample reported having been tested in the past year is disconcerting. This estimate is considerably lower than the 2014 NHBS wherein more than two-thirds of GBMSM interviewed had been tested for HIV in the preceding 12 months [6]. Additionally, more than 1 in 10 participants in our study reported never having been tested, highlighting the need to promote novel and effective approaches to increase prevention service utilization in this community.

Our findings suggest that increasing GBMSM’s detailed knowledge about HIV transmission dynamics (including common modes of spread and per-act transmission probabilities) might help improve their testing behavior. Practically, this task may be achieved by encouraging them to access publicly available information on CDC’s HIV website and other federal Web-based HIV resources. From 2005 to 2014, black or African American and Hispanic GBMSM aged 13-24 years saw the steepest increases in HIV diagnoses of approximately 87% [1]. Given that our younger, racial and ethnic minority participants were significantly less knowledgeable about HIV transmission dynamics, targeting these subgroups could potentially yield substantial prevention benefits. Previous research with racial and ethnic minorities has found a correlation between subjective HIV knowledge and testing [28]. Furthermore, the variety of acceptable testing options now available to GBMSM (eg, rapid home self-testing, individual voluntary counseling and testing, couples’ HIV testing and counseling) could facilitate both initial and repeated testing [29]. Regular testing could in turn increase knowledge about different components of the HIV prevention tool kit, thereby helping GBMSM protect themselves and their partners [30].

Regarding high-risk sexual behavior, almost half of our participants reported having engaged in CAS in the past 3 months, more than a third of whom had 2 or more male partners. According to a recent meta-analysis, the risk of HIV acquisition through sexual contact is the greatest for receptive CAS, ranging from 102 to 186 per 10,000 exposures [31]. Therefore, it is a cause for concern that more than a third of our sample had engaged in receptive CAS in the past 3 months. Besides HIV, CAS also increases the risk of other sexually transmitted diseases such as chlamydia, gonorrhea, and syphilis [32]. Consistent PrEP use has been demonstrated to reduce the risk of HIV infection among GBMSM by up to 92% [10] but fails to offer protection against other sexually transmitted diseases. Although our survey did inquire about current PrEP use, only 254 (24%) participants responded to that particular question, 12 (5%) of whom reported being on PrEP. This estimate is similar to the national prevalence of PrEP use among GBMSM in the United States [6].

Our study did not find an association between increasing levels of detailed knowledge about HIV epidemiology or transmission dynamics and engaging in CAS in the past 3 months. This contrasts with previous research among GBMSM that has linked greater knowledge concerning HIV with safer sexual practices [33,34]. Given that those studies used a true-false format to assess basic knowledge about HIV and associated risk factors, our findings suggest that a greater awareness of specific factual details might not be necessary to effect risk reduction. Another explanation for our null result could be that theoretical constructs other than information, such as motivation to practice preventive behaviors, might be more influential in reducing high-risk sexual behavior. The information-motivation-behavioral skills model states that information and motivation could be independent of each other, as observed when well-informed individuals are not motivated to change their HIV risk behavior or when persons who are motivated to practice preventive behaviors are not particularly well informed [35]. A relatively recent study with 391 at-risk GBMSM found that self-rated motivation was a significant predictor of CAS, as were behavioral skills such as keeping condoms nearby, reducing the number of sexual partners, and discussing safer sex with a partner [36].

### Strengths and Limitations

Strengths of our study include evaluating associations of detailed knowledge pertaining to the separate domains of HIV epidemiology and transmission dynamics with preventive and risky sexual behaviors in a large sample of GBMSM. Participants were recruited through the Web in a time-, cost-, and resource-efficient manner and represent sexually active GBMSM from all geographic regions of the United States. Because our survey was entirely voluntary and could only be accessed by clicking on our banner advertisements, it is unlikely that the same individual would have responded more than once. The perceived anonymity of a Web-based environment contributes to a greater honesty in reporting sensitive information, thereby reducing the possibility of social desirability bias [37]. Considering that the Web has become an increasingly popular venue among GBMSM to access sexual health information, including HIV testing resources [38-40], and to negotiate both high-risk and safe sex [41-43], we believe that understanding the relationships between these issues is critical in advancing Web-based HIV prevention efforts targeting members of this community.

However, we acknowledge there are several limitations to our study. Caution must be exercised in generalizing results to users of other social networking websites, all Web-using GBMSM, and those in the general US population. Our convenience sampling process yielded a group that was older and predominantly non-Hispanic white, characteristics that do not typically reflect persons at highest risk of acquiring HIV. Nevertheless, the low levels of testing and the high prevalence of CAS observed in our study highlight the need to engage all GBMSM in future comprehensive HIV prevention programs. Although it is surprising that the age distribution was skewed away from younger individuals, the unfortunate underrepresentation of racial and ethnic minority GBMSM in our sample is analogous to previous Web-based research studies [44]. Reduced access to and the use of both basic and high-speed Web services in this demographic may explain this disparity [45]. Finally, the cross-sectional nature of our data precludes drawing firm conclusions about the temporality of the association between detailed HIV transmission dynamics knowledge and testing history.

### Conclusions

Despite these limitations, our study provides preliminary evidence regarding whether and how different knowledge domains relate to preventive and high-risk sexual behaviors among GBMSM. Increasing detailed knowledge about HIV epidemiology might not be as important as educating those who are sexually active regarding transmission dynamics. Researchers and practitioners designing public health messages targeting GBMSM should bear in mind that not all knowledge is equal and that some aspects might have a greater positive impact than others. Future research to identify more influential content and contemporary modes of delivery is needed to generate and disseminate effective HIV prevention messaging.

## Appendix

Multimedia Appendix 1 List of 9 questions with response options assessing detailed knowledge about HIV epidemiology.

Multimedia Appendix 2 List of 8 questions with response options assessing detailed knowledge about HIV transmission dynamics.

## Abbreviations

CAS: condomless anal sex
CDC: Centers for Disease Control and Prevention
GBMSM: gay, bisexual, and other men who have sex with men
HIV: human immunodeficiency virus
NHBS: National HIV Behavioral Surveillance
PrEP: preexposure prophylaxis
TasP: treatment as prevention

## References

[1] (2016). CDC.

[2] (2015). CDC.

[3] Rosenberg ES, Grey JA, Sanchez TH, Sullivan PS (2016). Rates of prevalent HIV infection, prevalent diagnoses, and new diagnoses among men who have sex with men in US states, metropolitan statistical areas, and counties, 2012-2013. JMIR Public Health Surveill.

[4] (2015). AIDS.

[5] Cohen MS, Chen YQ, McCauley M, Gamble T, Hosseinipour MC, Kumarasamy N, Hakim JG, Kumwenda J, Grinsztejn B, Pilotto JH, Godbole SV, Mehendale S, Chariyalertsak S, Santos BR, Mayer KH, Hoffman IF, Eshleman SH, Piwowar-Manning E, Wang L, Makhema J, Mills LA, de Bruyn G, Sanne I, Eron J, Gallant J, Havlir D, Swindells S, Ribaudo H, Elharrar V, Burns D, Taha TE, Nielsen-Saines K, Celentano D, Essex M, Fleming TR, HPTN 052 Study Team (2011). Prevention of HIV-1 infection with early antiretroviral therapy. N Engl J Med.

[6] (2016). CDC.

[7] Jenness S, Goodreau S, Rosenberg E, Beylerian E, Hoover K, Smith D (2016). Impact of the Centers for Disease Control's HIV preexposure prophylaxis guidelines for men who have sex with men in the United States. J Infect Dis.

[8] Cohen MS, McCauley M, Gamble TR (2012). HIV treatment as prevention and HPTN 052. Curr Opin HIV AIDS.

[9] Beer L, Bradley H, Mattson CL, Johnson CH, Hoots B, Shouse RL, Medical Monitoring Project (2016). Trends in racial and ethnic disparities in antiretroviral therapy prescription and viral suppression in the United States, 2009-2013: Trends in disparities in HIV care. J Acquir Immune Defic Syndr.

[10] Grant RM, Lama JR, Anderson PL, McMahan V, Liu AY, Vargas L, Goicochea P, Casapía M, Guanira-Carranza JV, Ramirez-Cardich ME, Montoya-Herrera O, Fernández T, Veloso VG, Buchbinder SP, Chariyalertsak S, Schechter M, Bekker L, Mayer KH, Kallás EG, Amico KR, Mulligan K, Bushman LR, Hance RJ, Ganoza C, Defechereux P, Postle B, Wang F, McConnell JJ, Zheng J, Lee J, Rooney JF, Jaffe HS, Martinez AI, Burns DN, Glidden DV, iPrEx ST (2010). Preexposure chemoprophylaxis for HIV prevention in men who have sex with men. N Engl J Med.

[11] Branson BM, Handsfield HH, Lampe MA, Janssen RS, Taylor AW, Lyss SB, Clark JE, Centers for Disease Control and Prevention (CDC) (2006). Revised recommendations for HIV testing of adults, adolescents, and pregnant women in health-care settings. MMWR Recomm Rep.

[12] Paz-Bailey G, Hall I, Wolitski RJ, Prejean J, Van Handel MM, Le B, LaFlam M, Koenig L, Mendoza M, Rose RE, Valleroy L (2013). HIV testing and risk behaviors among gay, bisexual, and other men who have sex with men - United States. MMWR Morb Mortal Wkly Rep.

[13] Fisher JD, Fisher WA (1992). Changing AIDS-risk behavior. Psychol Bull.

[14] Catania J, Kegeles S, Coates T (1990). Towards an understanding of risk behavior: an AIDS risk reduction model (ARRM). Health Educ Q.

[15] Prochaska JO, Redding CA, Harlow LL, Rossi JS, Velicer WF (1994). The transtheoretical model of change and HIV prevention: a review. Health Educ Q.

[16] Rosenstock I, Strecher V, Becker M (1994). The health belief model and HIV risk behavior change.

[17] Bandura A (1994). Social cognitive theory and exercise of control over HIV infection.

[18] Sabaté E (2003). Adherence to long-term therapies: Evidence for action.

[19] (2002). UN.

[20] Sonenstein FL, Pleck JH, Ku LC (1989). Sexual activity, condom use and AIDS awareness among adolescent males. Fam Plann Perspect.

[21] Kelly JA, St Lawrence JS, Hood HV, Brasfield TL (1989). An objective test of AIDS risk behavior knowledge: scale development, validation, and norms. J Behav Ther Exp Psychiatry.

[22] Goh D (1993). The development and reliabilty of the Attitudes Toward AIDS Scale. Coll Stud J.

[23] Davis C, Sloan M, Macmaster S, Hughes L (2007). The International AIDS Questionnaire - English version (IAQ-E): assessing the validity and reliability. J HIV AIDS Prev Child Youth.

[24] Carey MP, Morrison-Beedy D, Johnson BT (1997). The HIV-Knowledge Questionnaire: development and evaluation of a reliable, valid, and practical self-administered questionnaire. AIDS Behav.

[25] Carey MP, Schroder KE (2002). Development and psychometric evaluation of the brief HIV Knowledge Questionnaire. AIDS Educ Prev.

[26] Belsley D (1991). Conditioning diagnostics: Collinearity and weak data in regression. 1st ed.

[27] Workowski KA, Berman S, Centers for Disease Control and Prevention (CDC) (2010). Sexually transmitted diseases treatment guidelines, 2010. MMWR Recomm Rep.

[28] Phillips KA (1993). Factors associated with voluntary HIV testing for African-Americans and Hispanics. AIDS Educ Prev.

[29] Sharma A, Stephenson RB, White D, Sullivan PS (2014). Acceptability and intended usage preferences for six HIV testing options among internet-using men who have sex with men. Springerplus.

[30] (2016). CDC.

[31] Patel P, Borkowf CB, Brooks JT, Lasry A, Lansky A, Mermin J (2014). Estimating per-act HIV transmission risk: a systematic review. AIDS.

[32] (2016). CDC.

[33] Benotsch EG, Kalichman S, Cage M (2002). Men who have met sex partners via the Internet: prevalence, predictors, and implications for HIV prevention. Arch Sex Behav.

[34] Dilley JW, McFarland W, Sullivan P, Discepola M (1998). Psychosocial correlates of unprotected anal sex in a cohort of gay men attending an HIV-negative support group. AIDS Educ Prev.

[35] Fisher J, Fisher W, Peterson JL, DiClemente RJ (2000). Theoretical approaches to individual-level change in HIV risk behavior. Handbook of HIV Prevention.

[36] Kalichman SC, Picciano JF, Roffman RA (2008). Motivation to reduce HIV risk behaviors in the context of the Information, Motivation and Behavioral Skills (IMB) model of HIV prevention. J Health Psychol.

[37] Krumpal I (2013). Determinants of social desirability bias in sensitive surveys: a literature review. Qual Quant 2013/06/01.

[38] Mitchell KJ, Ybarra ML, Korchmaros JD, Kosciw JG (2014). Accessing sexual health information online: use, motivations and consequences for youth with different sexual orientations. Health Educ Res.

[39] Hooper S, Rosser BR, Horvath KJ, Oakes JM, Danilenko G, Men's INTernet Sex II (MINTS-II) Team (2008). An online needs assessment of a virtual community: what men who use the internet to seek sex with men want in Internet-based HIV prevention. AIDS Behav.

[40] Swendeman D, Rotheram-Borus MJ (2010). Innovation in sexually transmitted disease and HIV prevention: internet and mobile phone delivery vehicles for global diffusion. Curr Opin Psychiatry.

[41] Rosser BR, Oakes JM, Horvath KJ, Konstan JA, Danilenko GP, Peterson JL (2009). HIV sexual risk behavior by men who use the Internet to seek sex with men: results of the Men's INTernet Sex Study-II (MINTS-II). AIDS Behav.

[42] Horvath KJ, Oakes JM, Rosser BR (2008). Sexual negotiation and HIV serodisclosure among men who have sex with men with their online and offline partners. J Urban Health.

[43] Liau A, Millett G, Marks G (2006). Meta-analytic examination of online sex-seeking and sexual risk behavior among men who have sex with men. Sex Transm Dis.

[44] Sullivan PS, Khosropour CM, Luisi N, Amsden M, Coggia T, Wingood GM, DiClemente RJ (2011). Bias in online recruitment and retention of racial and ethnic minority men who have sex with men. J Med Internet Res.

[45] (2015). Pew Research Center.

