# Supplementary material for: Detailed Knowledge About HIV Epidemiology and Transmission Dynamics and Their Associations With Preventive and Risk Behaviors Among Gay, Bisexual, and Other Men Who Have Sex With Men in the United States
Source: JMIR Public Health Surveill. 2017 Mar 6;3(1):e11. doi: 10.2196/publichealth.7255 (PMC5359415; doi:10.2196/publichealth.7255)
Supplement: Multimedia Appendix 1 [file publichealth_v3i1e11_app1.pdf]

Detailed Knowledge Domain: HIV Epidemiology

1. Approximately how many people in the United States are newly infected with HIV each year?
   - 30,000
   - 40,000
   - 50,000
   - 60,000

2. Approximately how many people in the United States are currently living with HIV?
   - 500,000
   - 1.2 million
   - 3.5 million
   - 5 million

3. What percentage of all those living with HIV in the United States do not know that they are infected?
   - 5%
   - 14%
   - 38%
   - 51%

4. Even though men who have sex with men comprise only 2% of the United States population, what proportion of new HIV infections did they account for annually from 2008-2010?
   - 1/6
   - 1/2
   - 3/4
   - 5/6

5. Even though African Americans comprise only 12% of the United States population, what percentage of new HIV infections did they account for in 2010?
   - 8%
   - 22%
   - 44%
   - 69%

6. Even though young people aged 13-24 years comprise only 16% of the United States population, what percentage of new HIV infections did they account for in 2010?
   - 4%
   - 26%
   - 49%
   - 70%

7. Who had the highest percentage of newly identified HIV-positive test results in 2010?

- Cisgender men or those who identify with their assigned male sex at birth
- Cisgender women or those who identify with their assigned female sex at birth
- Transgender individuals or those who do not identify with their assigned sex at birth

8. Specifically among young people aged 13-24 years, what percentage of all new HIV infections did young men who have sex with men account for in 2010?

- 11%
- 34%
- 72%
- 91%

9. Among gay and bisexual men in 2013, _____ accounted for the largest estimated percentage of HIV diagnoses followed by _____ and _____.

- Whites, Hispanics or Latinos, African Americans
- Hispanics or Latinos, African Americans, Whites
- Whites, African Americans, Hispanics or Latinos
- African Americans, Whites, Hispanics or Latinos
