# Supplementary material for: Detailed Knowledge About HIV Epidemiology and Transmission Dynamics and Their Associations With Preventive and Risk Behaviors Among Gay, Bisexual, and Other Men Who Have Sex With Men in the United States
Source: JMIR Public Health Surveill. 2017 Mar 6;3(1):e11. doi: 10.2196/publichealth.7255 (PMC5359415; doi:10.2196/publichealth.7255)
Supplement: Multimedia Appendix 2 [file publichealth_v3i1e11_app2.pdf]

Detailed Knowledge Domain: HIV Transmission Dynamics
1. The estimated probability of HIV infection per-act of receptive anal intercourse (receiving the penis into the anus, also known as bottoming) without any means of prevention is _____ per 10,000 exposures.<br>• 0<br>• 24<br>• 67<br>• 138
2. The estimated probability of HIV infection per-act of insertive anal intercourse (inserting the penis into the anus, also known as topping) without any means of prevention is _____ per 10,000 exposures.<br>• 0<br>• 11<br>• 46<br>• 93
3. The estimated probability of HIV infection per-act of receptive vaginal intercourse (receiving the penis into the vagina) without any means of prevention is _____ per 10,000 exposures.<br>• 0<br>• 8<br>• 31<br>• 58
4. The estimated probability of HIV infection per-act of insertive vaginal intercourse (inserting the penis into the vagina) without any means of prevention is _____ per 10,000 exposures.<br>• 0<br>• 4<br>• 27<br>• 63
5. The estimated probability of acquiring HIV from kissing without any means of prevention is _____ per 10,000 exposures.<br>• 0<br>• 5<br>• 19<br>• 41
6. The estimated probability of acquiring HIV from needle-sharing during injection drug use without any means of prevention is _____ per 10,000 exposures.<br>• 0<br>• 22

- 44
- 63

7. The estimated probability of acquiring HIV from a mosquito bite without any means of prevention is ______ per 10,000 exposures.
- 0
- 3
- 19
- 37

8. What is the probability of acquiring HIV from receptive or insertive oral sex without using any means of prevention?
- Extremely low
- Moderate
- High
- Biologically impossible
